# Compartment-Specific Mitochondrial Proteomic Alterations in Rat Hippocampus Following Chronic Social Isolation Stress

**DOI:** 10.3390/ijms27083386

**Published:** 2026-04-09

**Authors:** Dragana Filipović

**Affiliations:** Department of Molecular Biology and Endocrinology, “VINČA”, Institute of Nuclear Sciences—National Institute of the Republic of Serbia, University of Belgrade, 11000 Belgrade, Serbia; dragana@vinca.rs

**Keywords:** non-synaptic mitochondria, synaptosomes, depression-like behaviors, proteomics, hippocampus

## Abstract

Chronic social isolation (CSIS) is a form of psychosocial stressor strongly associated with the development of depression. Preclinical studies demonstrated that CSIS induces behavioral phenotypes resembling human depression, including anhedonia, behavioral despair and anxiety. This review summarizes proteomic-driven discoveries characterizing hippocampal non-synaptic mitochondria (NSM) and synaptosomal fractions containing synaptic mitochondria from adult male rats exposed to six weeks of CSIS, an animal model of depression, compared to controls. The compartment-specific proteomic alterations reveal mechanisms underlying mitochondrial dysregulation, providing molecular insights into the depression-like phenotype. Hippocampal NSM exhibit changes in energy metabolism-related proteins, including components of the tricarboxylic acid cycle and oxidative phosphorylation, as well as mitochondrial transport proteins and alterations in chaperones, structural and translational proteins, and monoamine oxidase, further elucidating how these proteomic changes contribute to mitochondrial dysregulation. In contrast, synaptosomal proteomics reveal predominantly increased protein abundance associated with energy metabolism, signaling, cytoskeletal organization, protein quality control, and vesicle trafficking, suggesting compensatory adaptations. Together, these findings highlight compartment-specific mitochondrial proteomic changes that may underlie depression-like behaviors and represent potential targets for therapeutic intervention.

## 1. Introduction

Psychosocial stress represents a significant risk factor for major depressive disorder (MDD), commonly known as depression. Growing evidence indicates that stress-induced mitochondrial dysfunction can disrupt essential cellular processes, leading to synaptic dysfunction and contributing to the molecular and structural changes underlying depressive phenotypes. Such mitochondrial dysfunction includes impaired energy metabolism, compromised mitochondrial quality control, and altered dynamics, resulting in reduced ATP production, elevated oxidative stress, and impaired neuronal function [1,2,3]. Furthermore, mitochondria can interfere with intracellular signaling pathways [4], as mitochondrial ATP is essential for receptor-mediated signal transduction following neurotransmitter binding [5], vesicular trafficking, and neurotransmitter release at synaptic terminals [5,6,7]. Consequently, impairments in mitochondrial bioenergetics can contribute to reduced synaptic plasticity and overall neuronal dysfunction, both of which represent hallmarks of depression [8,9]. Additionally, dysfunctions of mitochondria-dependent processes may further disrupt brain circuits, affecting behavior and promoting pathological states associated with depression [10,11].

Chronic social isolation (CSIS), as a mild chronic stress, is a widely used and well validated model for investigating the underlying mechanisms of depression [12]. In this model, individually housed rats are deprived of any visual or tactile contacts with other animals, but have normal auditory and olfactory experiences [13]. This mild chronic stress induces depression-like behaviors in rats, including anhedonia, reflected by reduced sucrose preference, and behavioral despair, evidenced by increased immobility in forced swim tests [14,15]. These behavioral alterations are accompanied by significant changes in hippocampal mitochondrial protein abundance, as revealed by proteomic analyses [16,17]. Additionally, CSIS leads to oxidative damage and inflammatory alterations in the rat hippocampus [18].

Similarly, mild chromic stress paradigms demonstrate widespread mitochondrial dysfunction, characterized by disrupted mitochondrial ultrastructure, impaired oxidative phosphorylation (OXPHOS), and altered membrane potential across multiple brain regions, including the hippocampus, contributing to the energy deficits observed in depression [19]. Chronic mild unpredictable stress further demonstrates significant reductions in ATP content within both the prefrontal cortex and hippocampus [20,21]. Social defeat stress models corroborate these findings, showing reduced ATP levels in stress-sensitive brain regions alongside increased markers of oxidative damage, including elevated lipid peroxidation and protein carbonylation [22,23]. Consistent with preclinical findings, proteomic analyses of post-mortem brains from individuals with depression have revealed alterations in mitochondrial protein expression, with approximately 21% of mitochondrial proteins showing altered expression patterns [24,25]. Importantly, these metabolic and mitochondrial impairments often precede the onset of depression-like behaviors, suggesting a causal role for mitochondrial dysfunction in the development of depressive phenotypes and highlighting potential targets for therapeutic strategies.

## 2. Heterogeneity of Neuronal Mitochondria Across Subcellular Compartments

Mitochondria within neurons comprise a functionally diverse population characterized by distinct subcellular distributions, morphology features, and specialized proteomic profiles. These organelles exhibit dynamic mobility across neuronal compartments, including dendrites, the axon shaft, and pre-synaptic terminals [26], and can be categorized into non-synaptic and synaptic sub-populations, each adapted to meet specific bioenergetic and signaling requirements within different neuronal compartments [7,27,28,29].

Non-synaptic mitochondria (NSM) reside primarily in the neuronal soma, axonal segments, and non-neuronal brain cells (such as glia, and endothelial), where they support basal bioenergetic demands and maintain redox balance, generating ATP required for the cellular processes in post-synaptic compartment [24]. Beyond their role in energy production, NSM mediate mitochondrial quality control, participating in fusion–fission dynamics, mitophagy-mediated clearance of damaged organelles, and regulation of intrinsic apoptotic signaling cascades. They are enriched in mitophagy receptors which coordinate the selective degradation of dysfunctional mitochondria, limiting accumulation of reactive oxygen species (ROS) and pro-apoptotic factors [30]. Importantly, modulation of protein expression levels within NSM represents a key mechanism through which neurons respond to chronic stress conditions.

Synaptic mitochondria are localized to the pre-synaptic compartment, where they provide ATP for maintaining synaptic homeostasis, neurotransmitter release, and pre-synaptic mechanisms underlying drug actions [24,31]. This local ATP is essential for both exocytosis and endocytosis as well as neurotransmitters reuptake [6,32]. Synthesized in the neuronal soma and transported along axons or dendrites to the pre-synaptic terminal [33], synaptic mitochondria are often “older” than somatic or glial mitochondria and accumulate higher levels of oxidative damage over time [34]. Isolated from synaptosomes [35], alterations in their proteome composition can compromise active zone function and impair synaptic transmission by affecting calcium buffering, ATP availability, and synaptic vesicle recycling [36].

Synaptic and NSM differ substantially across multiple parameters, including morphology [36,37], proteomic composition [38], enzymatic activity [36], calcium handling capacity [27], and susceptibility to oxidative stress [39], reflecting their adaptation to local cellular demands and accounting for their differential susceptibility to stress-induced dysfunction.

Table 1 summarizes these differences, emphasizing the functional heterogeneity of neuronal mitochondria and susceptibility of synaptic mitochondria compared to NSM.

A critical question in understanding the mitochondrial contribution to depression pathophysiology is whether NSM and synaptic mitochondria exhibit distinct alterations in protein composition and abundance under stress conditions. This review examines proteomic analyses of NSM and synaptosomes containing synaptic mitochondria isolated from the hippocampus of adult male Wistar rats exposed to six weeks of social isolation, an established animal model of depression, and compared to group-housed control animals. NSM and synaptosomes were prepared following Kristian’s differential centrifugation protocol [51]. Proteomic analyses were performed using a multi-step workflow: proteins were separated by SDS-PAGE, followed by in-gel tryptic digestion of excised bands and identification via liquid chromatography–tandem mass spectrometry (LC-MS/MS) on a Dionex RSLC nano HPLC system coupled to a Linear Trap Quadrupole Orbitrap XL mass spectrometer. Relative protein quantification between experimental groups was achieved through label-free quantification (LFQ) using PEAKS Studio 7.5 software (Bioinformatics Solutions Inc.) and Sieve 2.0 software (Thermo Fisher Scientific). Protein–protein interaction networks were constructed for all significantly regulated proteins in both mitochondrial subfractions (fold change > 1.5 or <0.8) using UniProtKB accession numbers and the STRING database (version 11.0) [16,17] (Supplementary Tables S1 and S2).

The hippocampus was chosen for this study due to its central role in learning and memory [52] and its involvement in regulating emotion, stress, and the reward system [53,54,55]. Neuroimaging studies in both patients and stress-induced animal models of depression or anxiety have revealed significant reductions in both hippocampal volume and dendritic spine density [56]. Chronic stress impairs hippocampal neurogenesis and neuroplasticity [57], contributing substantially to the cognitive and emotional deficits characteristic of depressive and anxiety disorders [58]. Importantly, animal models of depression reveal marked disturbances in hippocampal energy metabolism, making it particularly vulnerable to chronic stress [59]. These features collectively position the hippocampus as an ideal locus for investigating mitochondrial dysfunction in the context of stress-related psychiatric disorders.

To the best of my knowledge, no previous study has specifically examined the proteome of the rat brain hippocampal NSM and synaptosomes containing synaptic mitochondria in the context of CSIS-induced depressive-like behavior. The present findings therefore provide novel insight into the compartment-specific dysregulation of mitochondrial metabolism, which may remain undetectable in bulk tissue or unfractionated mitochondria analyses.

## 3. CSIS-Associated Changes in the Hippocampal NSM Proteome

### 3.1. Pyruvate Metabolism and TCA Cycle Alterations in NSM

**Figure 1:**
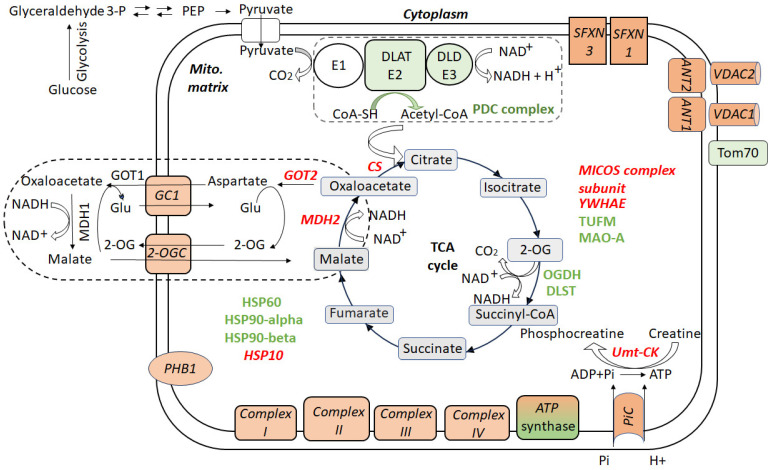
Schematic overview of redox metabolism and associated protein-level changes in hippocampal non-synaptic mitochondria (NSM) of rats subjected to chronic social isolation (CSIS) that showed depression-like behaviors. Proteins with elevated levels are shown in green, whereas those with reduced levels are shown in red and italicized. Abbreviations: 10 kDa heat shock protein (HSP10); 14-3-3 protein epsilon (YWHAE); 2-oxoglutarate dehydrogenase (2-OG); 2-oxoglutarate dehydrogenase complex component E1 (OGDH); 2-oxoglutarate/malate carrier protein (OGC); 60 kDa heat shock protein, (HSP60); 90 kDa heat shock protein alpha and beta (HSP90 alpha and HSP90 beta); adenine nucleotide translocase 1 and 2 (ANT1 and ANT2); citrate synthase (CS); Complex I (NADH:ubiquinone oxidoreductase); Complex II (succinate:ubiquinone oxidoreductase); Complex III (ubiquinol:cytochrome c oxidoreductase); Complex IV (cytochrome c oxidase, COX); Complex V (ATP synthase); glutamate (Glu); dihydrolipoyl dehydrogenase (DLD) (E3 component of PDC); dihydrolipoyllysine-residue succinyltransferase (DLST); malate dehydrogenase 1 (MDH1); malate dehydrogenase 2 (MDH2); mitochondrial contact site and cristae organizing system (MICOS complex subunit); mitochondrial creatine kinase, ubiquitous (Umt-CK); mitochondrial elongation factor Tu (TUFM); mitochondrial glutamate carrier (GC1); mitochondrial phosphate carrier (PiC); monoamine oxidase A (MAO-A); oxidative phosphorylation (OXPHOS); phosphoenolpyruvate (PEP); prohibitin 1 (PHB1); pyruvate dehydrogenase complex (PDC); pyruvate dehydrogenase E1 component, dihydrolipoyllysine-residue acetyltransferase (DLAT) (E2 component of PDC); sideroflexin 1 and 3 (SFXN1 and SFXN3); translocase of the outer mitochondrial membrane 70 kDa subunit (TOM70); voltage-dependent anion channel 1 and 2 (VDAC1 and VDAC2).

Proteomic analysis of the hippocampal NSM from CSIS-exposed rats revealed altered protein levels of enzymes involved in the pyruvate metabolism and TCA cycle compared to controls [16]. Proteins of the pyruvate dehydrogenase (PDH) complex were upregulated, including dihydrolipoyllysine-residue acetyltransferase (DLAT), the E2 catalytic acetyltransferase subunit, and dihydrolipoyl dehydrogenase (DLD), the E3 component which oxidizes the reduced lipoyl moieties of E2 and regenerates NADH [60] (Figure 1). This coordinated upregulation suggests increased pyruvate entry into the TCA cycle via its oxidative decarboxylation to acetyl-CoA potentially reflecting a compensatory metabolic response to elevated energetic demands imposed by CSIS.

In contrast, several enzymes of the TCA cycle exhibited reduced protein levels, including citrate synthase (CS), malate dehydrogenase (MDH2), and aspartate aminotransferase, mitochondrial (GOT2). CS functions as a rate-limiting enzyme that catalyzes the first committed step of the TCA cycle, while MDH2 and GOT2 participate in linking the TCA cycle with amino acid metabolism and the malate–aspartate shuttle. Their decreased abundance indicates compromised TCA function and restricted cycle flux, potentially limiting ATP production and impairing mitochondrial redox shuttling. Such metabolic perturbations are consistent with disrupted neuronal bioenergetics and may contribute to the behavioral manifestations of CSIS-induced depression-like phenotypes. Indeed, mitochondrial dysfunction in depression has been extensively documented, with evidence of reduced ATP synthesis and dysregulated mitochondrial dynamics in both clinical and preclinical studies [61,62]. Notably, NSM, which play an essential role in sustaining cellular energy homeostasis, appear particularly vulnerable to chronic stress-induced metabolic impairment [19].

Interestingly, the only TCA cycle-associated proteins displaying increased expression were subunits of the 2-oxoglutarate dehydrogenase (2-OG) complex, specifically dihydrolipoamide S-succinyltransferase (DLST, E2) and 2-oxoglutarate dehydrogenase (OGDH, E1), which catalyze the conversion of 2-oxoglutarate to succinyl-CoA (Figure 1) [63]. These enzymes also represent critical nodes for regulating 2-OG flux toward amino acid biosynthetic pathways [64]. Although pyruvate was directed into the TCA cycle, the downregulation of other TCA cycle components indicates incomplete metabolic adaptation and energy insufficiency in hippocampal neurons subjected to CSIS.

Although proteomic studies have reported alterations in pyruvate metabolism and TCA cycle enzymes in animal models of depression, these investigations were predominantly performed on whole brain/brain region lysates without subcellular fractionation (Table 2). The observed discrepancies between pyruvate metabolism and TCA cycle alterations in NSM of CSIS rats and the reported literature may be attributed to differences in stress paradigms, the specific brain mitochondrial fractions analyzed (total mitochondria vs. NSM), species or strain (rat vs. mouse), age, and methodological variations in proteomics approaches.

### 3.2. Alterations in the NSM Electron Transport Chain and ATP Synthase

OXPHOS represents the primary source of cellular ATP generation. Clinical studies have demonstrated that patients with depression exhibit reduced mitochondrial ATP capacity relative to healthy controls [1,5,76]. Proteomic profiling of hippocampal NSM from CSIS rats revealed widespread dysregulation across all five OXPHOS complexes (Figure 1), indicating substantial disruption of mitochondrial bioenergetics. Multiple subunits of NADH dehydrogenase (ubiquinone) or Complex I showed significant downregulation, including NDUFB10, NDUFS2, NDUFA13, NDUFB5, NDUFA2, NDUFV2, and NDUFS3 (Supplementary Table S1). As the largest component and entry point of the electron transport chain (ETC), Complex I mediates electron transfer from NADH to ubiquinone, establishing the proton gradient required for ATP synthesis [77]. The coordinated downregulation of multiple Complex I subunits in NSM of CSIS rats suggests potential trends toward diminished ETC capacity and reduced NADH oxidation efficiency, but does not directly indicate compromised functional outcomes such as ATP production or ROS generation. Impaired Complex I activity has been linked to stress-induced metabolic reprogramming in preclinical models [78]. Additionally, in post-mortem brains of MDD patients, subunits NDUFV1, NDUFV2, and NDUFS1 showed decreased protein levels [79,80]. Several mitochondrial Complex I subunits identified in NSM of CSIS rats are consistent with previous brain mitochondrial proteomics studies reporting altered protein expression of NDUFS2, NDUFA13, NDUFA2, NDUFV2, NDUFS3 in mitochondrial fractions from the (frontal) cortex and hippocampus, highlighting mitochondrial respiratory chain dysfunction as a common feature of brain pathology (Table 3).

Proteomic analysis further revealed downregulation of succinate dehydrogenase (SDHB) or Complex II and cytochrome c components of Complex III, including cytochrome c1 (CYC1) and cytochrome b-c1 complex subunit Rieske (UQCRFS1), in hippocampal NSM from CSIS rats. Both complexes utilize ubiquinone as the electron carrier: Complex II reduces ubiquinone to ubiquinol, which subsequently transfers electrons to Complex III to regenerate ubiquinone [81]. The concurrent reduction in abundance of both Complex II and III components is particularly relevant, as it suggests that the alternative electron entry point into the ETC, independent of Complex I, may also be compromised, thereby limiting the capacity for compensatory electron through the ubiquinone pool, a critical step for the efficient coupling of the TCA cycle to OXPHOS. However, it should be noted that these observations reflect changes in protein abundance rather than directly measured functional outcomes; whether they translate into impaired electron flow, increased electron leakage, or mitochondrial inefficiency will require functional validation [82]. Notably, both Complex I and III have been significantly decreased in the neuron-derived extracellular vesicles of MDD patients, providing translational relevance to the present proteomic findings [83].

Cytochrome c oxidase (COX) or Complex IV showed selective modulation of their subunits, with moderate reductions in COX4I1 and COX6B1 and more pronounced decreases in COX5B, while COX2 and COX5A showed the most severe suppression in NSM of CSIS rats, suggesting a coordinated impairment of Complex IV assembly and electron transfer capacity. Notably, among the 13 polypeptides encoded by mitochondrial DNA, only COX2 exhibited significant downregulation, whereas seven subunits of Complex I appeared to maintain normal synthesis. This selective vulnerability of COX2 may arise from several converging factors. Unlike many Complex I subunits, COX2 requires a specialized set of assembly factors, including COX20, synthesis of cytochrome c oxidase 1 and 2 (SCO1 and SCO2), and cytochrome c oxidase assembly factor 6 (COA6), for the proper incorporation of copper into its CuA catalytic site, making it particularly sensitive to disturbances in copper homeostasis or assembly factor availability [84,85]. Furthermore, since COX1 serves as the nucleation core of Complex IV assembly, any newly synthesized COX2 that fails to associate with the COX1-containing assembly intermediate is rapidly targeted for degradation by mitochondrial AAA proteases [86]. The biogenesis of COX2 is further complicated by its dependence on the sequential action of COX20, which stabilizes its N-terminal transmembrane region, and COX18, which acts as a transient insertase, facilitating the translocation of the C-terminal domain containing the apo-CuA site across the inner mitochondrial membrane [87]. Together, these factors create a uniquely narrow biogenetic window for COX2 maturation, in contrast to the Complex I ND subunits, which appear to maintain normal translational output under the same conditions. Given its key role in facilitating electron transfer to oxygen at the terminal step of the ETC, reduced COX2 abundance may directly compromise electron transfer efficiency, OXPHOS, and diminish ATP production. Therefore, changes in COX2 levels may indicate impaired Complex IV assembly and point toward mitochondrial respiratory dysfunction. Specifically, downregulation of ETC Complexes I and IV at the protein level aligns with observations in MDD and other mood disorders, indicating that impaired ETC activity can reduce ATP production, disturb cellular redox balance, and increase oxidative stress in neurons [80]. Brain studies have demonstrated variable alterations in COX activity, with decreases in specific activity ranging from 0% to 80%, depending on the brain region and experimental model [88].

ATP synthase (Complex V) is the terminal enzyme of OXPHOS, responsible for the production of the majority of cellular ATP. In NSM of CSIS model, ATP synthase (Complex V) displayed a heterogeneous pattern of subunit regulation: ATP5F1A and ATP5F1B were upregulated, whereas ATP5F1C and ATP5H were slightly downregulated, and ATP5PB and ATP5ME were more substantially reduced [16]. This mixed pattern suggests a complex remodeling of the ATP synthase machinery, potentially reflecting incomplete compensatory adaptations aimed at sustaining energy production under CSIS. The selective upregulation of specific subunits likely represents a cellular attempt to compensate for diminished efficiency elsewhere in the respiratory chain, underscoring the dynamic interplay between mitochondrial complex protein expression and neuronal energy homeostasis under CSIS.

Mitochondrial dysfunction, characterized by disrupted OXPHOS and impaired energy metabolism, has been increasingly implicated in the pathophysiology of depression, correlating with decreased ATP availability, increased ROS, and compromised neuronal function [61]. Moreover, ATP levels are decreased in key brain regions after social isolation or other stress procedures used as depression models in rodents. This decrease is consistently seen in the hippocampus, prefrontal cortex (PFC), and nucleus accumbens (NAc), which are all regions involved in emotion and motivation [89]. Moreover, quantitative data may vary depending on the model and experimental procedures; data confirm a drop in ATP in relevant brain structures in stress/depression models [89,90,91]. Proteomic investigations employing diverse animal models of depression have identified alterations in OXPHOS-associated proteins (Table 3).

Additionally, ubiquitous mitochondrial creatine kinase (Umt-CK), a key enzyme critical for energy buffering, was also downregulated in hippocampal NSM from CSIS rats (Figure 1). Umt-CK catalyzes the reversible transfer of high-energy phosphates from ATP to creatine, forming phosphocreatine and ADP, which enables rapid ATP regeneration and efficient energy transfer in neurons [92]. Its reduced abundance implies a diminished capacity for mitochondrial energy buffering and redistribution during periods of high metabolic demand, potentially limiting ATP availability and increasing neuronal vulnerability to stress.

**Table 3:** Evidence of mitochondrial respiratory chain subunit dysregulation across animal models and human post-mortem studies of stress, depression, and anxiety-related disorders.

Respiratory Complex	Protein	Stress Model/ Brain Region	Expression Change	Technique	Key Finding	References
Complex I (NADH dehydrogenase)	NDUFS1/NDUFS3	Sleep deprivation; mouse; hippocampus	↓	WB	Reduced NDUFS3 and decreased Complex I activity	[93]
	NDUFS1/UQCRFS1	CSIS; rat; PFC; NSM	↑	LC-MS/MS	Adaptive mitochondrial response to increased energy demand	[15]
	NDUFA2/NDUFS3/NDUFA13	AD; human brain frontal cortex mitochondria; late-onset	↓	iTRAQ-based proteomics	Downregulation of subunits in the late-onset AD	[94]
	NDUFA13	Human MDD; DLPFC	↑	1D-LC-MS	Altered mitochondrial Complex I function and disrupted brain energy metabolism associated with depression and psychotic symptoms	[95]
	NDUFA2	AD; human brain frontal cortex mitochondria; early-onset	↓	iTRAQ-based proteomics	Significant reduction in Complex I subunit in early-onset AD	[94]
	NDUFA2/NDUFV3	CMS; rat; cerebellum	↓	iTRAQ-based proteomics	Downregulation linked to mitochondrial dysfunction	[71]
	NDUFV1/NDUFS1/NDUFV2	Schizophrenia and PPD; human brain	↑	RT-qPCR	NDUFV2 upregulated in schizophrenia—opposite to depression findings	[96]
	NDUFV2/NDUFS2/NDUFS3	CMS; rat; hippocampal synaptosomes	↓/↑/↑	iTRAQ-based proteomics	Abnormal activity of hippocampal synaptic mitochondria-related OXPHOS pathways in CMS susceptible rats	[97]
	NDUFS3	CMS; rat; whole hippocampal lysate (Dep-Sus/Anx-Sus/Insus)	↑/↑/↔	iTRAQ-based proteomics	Dep-sus ↑; Anx-sus ↑; Insus ↔	[69]
	NDUFS3	STZ-induced type-1 diabetic rat; forebrain mitochondria	↓	2D-HPLC-ESI-MS/MS	Reduced catalytic activity of Complex I	[98]
Complex II (Succinate dehydrogenase)	SDHB	HAB/LAB mouse lines; cingulate cortex; synaptosomes	↑	LC-MS/MS	↑ HAB/LAB ratio contributes to pathophysiology of anxious phenotype in HAB mice	[99]
	SDHB	SI-induced anxiety; mouse; hippocampal mitochondria	↓	Enzyme activity	~52% decrease in SDHB activity vs. control	[100]
Complex III (Cytochrome bc1)	CYC1	Rat zinc-deficiency depression-like model; PFC	↑	LC-MS/MS	Impaired mitochondrial respiration and energy metabolism in models of depression and zinc deficiency	[101]
	UQCRFS1/CYC1	HAB/LAB mouse lines; cingulate cortex synaptosomes	↑	LC-MS/MS	Upregulation of OXPHOS complexes as a general characteristic of the anxious brain	[99]
Complex IV (Cytochrome c oxidase)	MT-CO2 (COX2)	CMS; rat; cerebellum	↑	iTRAQ-based proteomics	Upregulation linked to mitochondrial respiratory chain disruption	[71]
	COX4I1/COX5A	CMS; hippocampal synaptosomes	↑	iTRAQ-based proteomics	Increased expression in CMS-susceptible animals	[97]
	COX4I1/COX5B	Human MDD; DLPFC	↑	1D SDS-LC-MS	An attempt by mitochondria to compensate for respiratory chain dysfunction caused by oxidative stress	[95]
	COX4I1/COX2	Rat zinc-deficiency depression-like model; PFC	↑/↑	LC-MS/MS	Adaptive response of neurons to maintain energy metabolism under zinc deficiency	[101]
	COX5A/COX5B	CMS; rat; hippocampus	↑	iTRAQ-based proteomics	Increased in stress-resilient rats as part of a stress-protection mechanism	[102]
	COX5A	Schizophrenia; post-mortem frontal cortex	↓	LC-MS	Reduced mitochondrial oxidative respiration	[103]
	COX6B1	I/R; rat; hippocampal neurons	↑	WB	Protection of hippocampal neurons from I/R-induced injury by enhancing Complex IV function and reducing apoptosis	[104]
	Complex I/II/IV	SI; mouse; PFC lysate	↓	WB; enzyme activity, ELISA	Changes in ETC indicate impaired mitochondrial energy metabolism	[105]
	ATP5F1B/ATP5F1D	CSIS-resilient vs. control rats; PFC; synaptosomes	↓	LC-MS/MS	Downregulation suggests diminished mitochondrial ATP synthesis capacity in resilient rats	[106]
Complex V (ATP synthase)	ATP5F1A/ATP5F1B	CSIS-resilient vs. CSIS-susceptible rats; NSM; hippocampus	↑	1D-LC-MS/MS	Strengthening OXPHOS capacity to support the high energy demands required for stress adaptation	[66,67]
	ATP5F1A	CSIS-resilient vs. control rats; NSM; hippocampus	↑	1D-LC-MS/MS	Improved energy supply	[66,67]
Energy Metabolism	ATP levels	SI; rat/mouse; NAc; hippocampus, PFC,	↓	Biochemical assays	Reduced ATP levels across brain regions	[90,100]

Literature findings of alterations in the protein expression, mRNA expression, and/or enzymatic activity of mitochondrial oxidative phosphorylation (OXPHOS) subunits across Complexes I–V, as identified by mass spectrometry-based proteomics (LC-MS/MS, iTRAQ, 1D-LC-MS, 2D-HPLC-ESI-MS/MS), western blot (WB), enzyme activity assays, and RT-qPCR. Data are derived from rodent models of chronic mild stress (CMS), chronic social isolation stress (CSIS), social isolation (SI); social isolation-induced anxiety, sleep deprivation, streptozotocin-induced diabetes, zinc deficiency, ischemia/reperfusion, high/low anxiety-related behavior (HAB/LAB) mouse lines, as well as human post-mortem brain tissue from patients with major depressive disorder (MDD), schizophrenia, paranoid personality disorder (PPD), and Alzheimer’s disease (AD). Brain regions examined include the prefrontal cortex (PFC), hippocampus, cerebellum, cingulate cortex, dorsolateral prefrontal cortex (DLPFC), and frontal cortex. Regulation is indicated as upregulation (↑), downregulation (↓), or no significant change (↔). Mixed regulation patterns across behavioral subgroups (depression-susceptible, Dep-Sus; anxiety-susceptible, Anx-Sus; stress-insusceptible, Insus) are indicated accordingly. NSM, non-synaptic mitochondria; ETC, electron transport chain; STZ, streptozotocin; NAc, nucleus accumbens; I/R, ischemia/reperfusion.

### 3.3. Alterations in NSM Transport Proteins

CSIS in hippocampal NSM induced substantial significant changes in the abundance of transport-related proteins in NSM, suggesting an altered capacity for metabolite exchange and altered energy homeostasis (Figure 1) (Supplementary Table S1). Notably, the mitochondrial import receptor subunit Tom70 (Tomm70) was upregulated. Tom70, located on the outer mitochondrial membrane, recruits chaperones (e.g., Hsp90, Hsp70) to facilitate the import of mitochondrial precursor proteins while protecting the cytosol from proteotoxic stress [107]. Its upregulation may represent an adaptive response aimed at enhancing the protein import capacity of NSM under CSIS.

In contrast, multiple transport-related proteins for mitochondrial–cytosolic metabolic coupling were downregulated. These included voltage-dependent anion channels (VDAC1 and VDAC2), transporters ADP/ATP translocases 1 and 2 (ANT1 and ANT2, encoded by genes Slc25a4 and Slc25a5, respectively) [80], the mitochondrial phosphate carrier (PiC, encoded by geneSlc25a3), the mitochondrial 2-oxoglutarate/malate carrier (2-OGC, encoded by Slc25a11), the mitochondrial glutamate carrier (GC1, encoded bySlc25a22), and sideroflexins (SFXN1, SFXN3). VDAC1 and VDAC2, located in the outer mitochondrial membrane, mediate the exchange of metabolites such as ATP/ADP and pyruvate, while also contributing to calcium homeostasis and apoptotic signaling. VDAC1, in particular, serves as a primary channel for nucleotide and metabolite exchange and interacts with regulatory proteins that influence mitochondrial function and cell survival [108]. ANT1 and ANT2, located in the inner mitochondrial membrane, facilitate ADP/ATP translocation, a process essential for maintaining cytosolic energy availability and proper mitochondrial homeostasis [109]. The combined downregulation of these transporters in hippocampal NSM of CSIS rats likely impairs mitochondrial–cytosolic metabolic coupling, limiting ATP supply to the cytosol, disrupting calcium signaling, and increasing susceptibility to oxidative stress. These alterations may compromise mitophagy and enhance apoptotic vulnerability, collectively contributing to depressive-like phenotypes under CSIS. Notably, hippocampal VDAC1 has been implicated in recognition memory. Studies have shown that both its downregulation in scopolamine-induced amnesic mice and its silencing in untreated mice are associated with mitochondrial dysfunction, reduced ATP production, increased oxidative stress, neuronal degeneration, and impaired memory [110].

2-OGC is an integral component of the malate–aspartate shuttle, enabling the transfer of reducing equivalents (from NADH) into the mitochondria by exchanging malate and α-ketoglutarate across the inner mitochondrial membrane [111]. Reduced abundance of hippocampal 2-OGC in CSIS rats may limit the mitochondrial–cytosolic exchange of 2-OG, potentially affecting TCA cycle flux and neuronal energy metabolism, whereas reduced GC1 levels could impair mitochondrial excitatory amino acid metabolism and disrupt glutamate neurotransmitter cycling. PiC was also reduced, potentially limiting inorganic phosphate import into the mitochondrial matrix, which would be expected to impair ATP synthesis and perturb calcium homeostasis. This reduction may further compromise Umt-CK, which was also found to be downregulated.

SFXN1 and SFXN3 are integral proteins of the mitochondrial inner membrane involved in amino acid transport. SFXN1 primarily mediates serine import into mitochondria, supporting one-carbon metabolism, and may also facilitate the transport of alanine, cysteine, and glycine, influencing mitochondrial metabolic flux and respiratory chain integrity [112]. SFXN3 similarly participates in mitochondrial amino acid transport, but is enriched in neurons and has been shown to modulate synaptic plasticity, suggesting a more prominent role in neuronal structure and function [113]. Reduced abundance of SFXN1 in hippocampal NSM of CSIS rats may impair mitochondrial one-carbon metabolism and energy balance, while decreased SFXN3 levels could disrupt mitochondrial amino acid handling and compromise neuronal and synaptic structure, potentially contributing to altered neuronal function. This coordinated impairment in hippocampal NSM transport capacity likely exacerbates bioenergetic insufficiency and increases neuronal vulnerability to CSIS, contributing to the depression-like behaviors.

The transport protein alterations identified in hippocampal NSM of CSIS rats are consistent with findings reported across multiple stress- and depression-related models. As summarized in Table 4, dysregulation of mitochondrial transport and carrier proteins, including VDAC isoforms, ANT1/ANT2, PiC, 2-OGC, GC1, and sideroflexins, has been documented across rodent models of chronic mild stress, chronic social defeat stress, zinc deficiency, and ischemia, as well as in human post-mortem brain tissue from patients with Alzheimer’s disease. Notably, alterations in several of these proteins have also been observed in hippocampal synaptosomal mitochondria under CMS conditions, indicating that stress-induced disruption of mitochondrial transport is not restricted to NSM but may extend across mitochondrial subpopulations and brain compartments. These findings underscore the vulnerability of mitochondrial metabolite exchange systems to chronic psychological stress and their potential contribution to the bioenergetic deficits underlying depressive-like phenotypes.

### 3.4. CSIS-Induced Changes in NSM Chaperones

Proteomic analysis revealed changes in chaperone protein levels in the hippocampal NSM of CSIS rats, indicating stress-induced remodeling of protein quality control systems. Among the mitochondrial chaperones, the mitochondrial 60 kDa heat shock protein (HSP60) was markedly upregulated, consistent with an elevated requirement for chaperonin-mediated folding and assembly of mitochondrial proteins, under conditions of mitochondrial stress and altered energy demand [119].

Moderate upregulation of heat shock proteins HSP90-alpha and HSP90-beta further suggests activation of cytoprotective pathways aimed at protein stabilization and stress signaling regulation, as HSP90 family members contribute to the maintenance of mitochondrial integrity and modulate protein turnover under stress [120]. Conversely, the small mitochondrial chaperone HSP10 was downregulated, undermining the functional interplay with HSP60 necessary for the efficient folding of mitochondrial matrix proteins. The HSP60–HSP10 chaperonin complex is essential for the correct folding of imported polypeptides and the maintenance of mitochondrial function, so perturbations in this system have been implicated in impaired mitochondrial proteostasis and neurodegenerative disorders [121]. Similarly, prohibitin 1 (PHB1), a multifunctional mitochondrial chaperone and structural protein involved in maintaining inner membrane integrity and facilitating protein stabilization, was reduced. Reduced prohibitins protein levels disrupt mitochondrial inner membrane architecture, enhances ROS generation, and increases susceptibility to apoptotic stimuli, effects that have been linked to abnormal mitochondrial morphology and neuronal dysfunction [122]. Moreover, a deficiency of prohibitins has been shown to induce mitochondrial fragmentation, resulting in reduced ATP production, aberrant mitochondrial morphology, protein aggregation, and neuronal death [123]. This imbalanced chaperone response, characterized by the upregulation of select chaperones alongside the downregulation of critical folding cofactors and structural chaperones, suggests incomplete or maladaptive proteostatic remodeling in hippocampal NSM under CSIS.

Evidence from other stress models and brain regions further supports the notion that chaperones are consistently dysregulated in depression-related conditions (Table 5). Across preclinical models, HSP60 downregulation has been reported in the hippocampus of social defeat stress mice [124], and coordinated reduction in both HSP60 and HSP10 has been observed in stress-resilient animals [124], suggesting that attenuation of chaperonin activity may paradoxically accompany adaptive outcomes under certain conditions. In clinical samples, elevated HSP90AA1 protein levels have been detected in the DLPFC of non-psychotic MDD patients [95], while HSP90AB1 mRNA upregulation has been reported in the hippocampus of CMS rats [125], collectively indicating that HSP90 isoform induction may represent a conserved stress-adaptive mechanism across species and brain regions.

### 3.5. Dysregulation of Structural and Translational Proteins in NSM

Changes in proteins related to mitochondrial structure and protein synthesis were also revealed in hippocampal NSM of CSIS rats. The MICOS complex subunit, a key component of the mitochondrial contact site and cristae organizing system, was downregulated. The MICOS complex is essential for maintaining cristae junctions and inner membrane architecture, and its disruption has been linked with aberrant cristae morphology and compromised mitochondrial structural integrity [128]. Thus, reduced protein levels of this subunit suggest impaired mitochondrial ultrastructure that may directly contribute to the observed deficits in energy metabolism under chronic stress.

In contrast, the mitochondrial elongation factor Tu (TUFM), which mediates the elongation phase of mitochondrial protein translation, was upregulated. Given that TUFM is critical for the biosynthesis of mitochondrially encoded subunits of OXPHOS complexes [129], its upregulation likely reflects a compensatory response to maintain mitochondrial protein synthesis under CSIS, supporting the production of essential components for OXPHOS despite structural deficits.

Additionally, 14-3-3 protein epsilon (YWHAE), a scaffolding protein involved in signal transduction and protein–protein interactions, was downregulated in hippocampal NSM of CSIS rats [16]. Members of the 14-3-3 protein family are known to regulate apoptotic signaling, mitochondrial dynamics, and stress-response pathways in neurons [129]. Reduced YWHAE protein levels may impair intracellular signaling pathways, potentially increasing vulnerability to CSIS. Furthermore, in the hippocampal synaptosomal fraction of CSIS-resilient versus CSIS-susceptible rats, downregulation of 14-3-3 beta/alpha/eta isoforms was observed [74], suggesting adaptive modulation of synaptic signaling that limits activation of stress-sensitive pathways and supports stress resilience. These findings suggest that CSIS induces a complex adaptive response in hippocampal NSM, attempting to balance structural deficits with enhanced translational capacity. While upregulation of mitochondrial translation machinery may partially compensate for structural and functional deficits, concurrent disturbances in signaling proteins imply that this adaptation may be insufficient, leaving neurons susceptible to bioenergetic and signaling dysfunction.

### 3.6. Increased MAO-A Protein Levels

Monoamine oxidase A (MAO-A) was significantly upregulated in NSM of CSIS rats compared to controls. As a key enzyme responsible for the oxidative deamination of monoamine neurotransmitters, including serotonin, dopamine, and norepinephrine, MAO-A plays a central role in mood regulation and stress responses [130]. Increased MAO-A protein levels suggest enhanced catabolism of these neurotransmitters, consistent with previous studies linking chronic stress and depressive-like behavior to reduced monoamine levels in the brain. In addition, MAO-A also catalyzes the degradation of certain biogenic amines and aldehydes, generating hydrogen peroxide as a byproduct of its enzymatic activity. Consequently, upregulation of MAO-A could contribute to increased oxidative stress within NSM, exacerbating mitochondrial dysfunction and metabolic disruption under CSIS. These findings support the hypothesis that CSIS modulates enzymatic pathways of monoaminergic metabolism, potentially disrupting neurotransmitter homeostasis and promoting depressive-like symptoms in chronically stressed rats. The observed increase in MAO-A protein expression may thus represent both a neurochemical and a mitochondrial mechanism linking chronic stress to depressive pathology. Moreover, MAO-A is upregulated across all three groups (depression-susceptible, anxiety-susceptible, and stress-resilient rats) in a rat model of chronic mild stress relative to controls [69], suggesting that enhanced monoamine catabolism represents a universal brain response to chronic mild stress, independent of the behavioral phenotype developed. In a social defeat stress model, repeated stress increased MAO-A gene expression in the raphe nuclei of the midbrain of mice, suggesting that aggressive social stress can upregulate MAO-A and contribute to altered serotonin metabolism linked to depressive- and anxiety-like behaviors [131]. In depressed humans, imaging studies measuring MAO-A binding density have confirmed higher MAO-A levels in regions like the prefrontal cortex and anterior cingulate cortex during major depressive episodes, reinforcing the idea that increased MAO-A is part of depression pathophysiology [132].

## 4. CSIS-Associated Changes in the Hippocampal Synaptosome Proteome

### 4.1. Adaptive Mitochondrial Responses in Synaptosomes: ATP8 and Chaperone Upregulation

**Figure 2:**
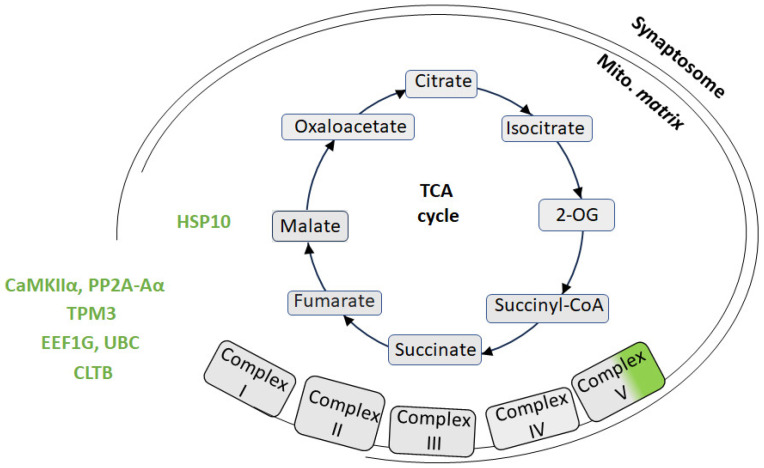
Schematic overview of protein level changes in the hippocampal synaptosome, including synaptosomal (synaptic) mitochondria of rats exposed to chronic social isolation (CSIS). Proteins with elevated levels are shown in green. Abbreviations: 2-oxoglutarate (2-OG); ATP synthase protein 8 (ATP8; calcium/calmodulin-dependent protein kinase type II subunit alpha (CaMKIIα); Isoform non-brain of clathrin light chain (CLTB); elongation factor 1-gamma (EEF1G); 10 kDa heat shock protein (HSP10); protein phosphatase 2 scaffold subunit A alpha (PP2A-Aα); tropomyosin alpha-3 chain (TPM3); polyubiquitin (UBC).

Proteomic analysis of synaptosomes from CSIS rats revealed upregulation of ATP synthase protein 8 (ATP8) [17] (Figure 2) (Supplementary Table S2), a mitochondrial-encoded subunit of the F_0_ component of the mitochondrial ATP synthase (Complex V). ATP8 contributes to the proton channel that allows H^+^ ions to flow through the F0 domain, driving ATP synthesis in the F1 domain [133]. This upregulation likely reflects increased energy demand at synapses, which is consistent with the need to maintain synaptic transmission and ionic homeostasis during stress conditions. Such a change may represent a compensatory mechanism to preserve ATP production, but it may also indicate reorganization or an imbalance in the function of Complex V, which is often associated with mitochondrial stress and neuropsychiatric disorders [1]. Chronic stress is known to profoundly affect neuronal mitochondrial function including changes to mitochondrial gene expression, dynamics, and energy metabolism [134]. The observed ATP8 upregulation may serve to meet the elevated energy requirements necessary for synaptic transmission, which accounts for approximately 75% of total brain energy consumption.

In parallel, the mitochondrial chaperone HSP10 was also upregulated in synaptosomes of CSIS rats. Acting as a co-chaperonin for HSP60, HSP10 facilitates the proper folding of imported mitochondrial proteins and helps prevent misfolding, promoting the correct assembly of polypeptides generated under stress in the mitochondrial matrix [135]. Its upregulation in synaptosomes suggests the activation of the mitochondrial protein quality control system (proteostasis) [136], likely in response to elevated oxidative stress and the accumulation of damaged proteins. This upregulation aligns with the mitochondrial unfolded protein response (UPRmt), a quality-control mechanism that prevents the accumulation of misfolded proteins [137]. While UPRmt has been extensively characterized in ischemic injury [138] and neurodegenerative disease models [139], its role in chronic psychological stress is less explored. Supporting this, Picard et al. [140] demonstrated that psychological stress accelerates mitochondrial aging and increases oxidative stress, conditions requiring enhanced chaperone activity. Chronic mild stress in animals induces depressive-like behaviors accompanied by reduced mitochondrial respiratory rates and dissipated mitochondrial membrane potential in the hippocampus [19], promoting protein misfolding and requiring increased chaperone function. Furthermore, proteomics analyses of the hippocampus from stressed animals further reveal significant alterations in mitochondrial proteins, including those involved in proteostasis [97]. Notably, in transgenic alpha-synuclein models, HSP10 has been associated with mitochondrial dysfunction in striatal synaptosomes [141], supporting its role in maintaining synaptic mitochondrial integrity under stress conditions. The coordinated upregulation of ATP8 and HSP10 suggests mitochondrial adaptation aimed at preserving both energy production capacity and proteostatic integrity within synaptic mitochondria, consistent with the concept of “mitochondrial allostatic load” under chronic stress [88].

### 4.2. Modulation of Synaptosome Kinases and Phosphatases

Proteomic profiling of CSIS rats’ synaptosomes revealed upregulation of both phosphatase 2 scaffold subunit A alpha (PP2A-Aα) and calcium/calmodulin-dependent protein kinase II alpha (CaMKIIα) (Supplementary Table S2). PP2A-Aα serves as a scaffold subunit of the PP2A holoenzyme, coordinating catalytic and regulatory subunits to regulate serine/threonine dephosphorylation of synaptic proteins, restoring synapses to their basal state. These cycles of phosphorylation and dephosphorylation provide a fundamental regulatory mechanism for learning, memory, and synaptic plasticity, with PP2A specifically implicated in fear memory consolidation [142]. Its increased protein expression suggests an enhancement of phosphatase-mediated modulation of synaptic signaling, which may act as a compensatory mechanism to maintain synaptic protein homeostasis under CSIS.

CaMKIIα is a pivotal synaptic kinase activated by calcium/calmodulin, mediating phosphorylation of post-synaptic receptors and other synaptic proteins critical for long-term potentiation (LTP) and synaptic plasticity [143]. Elevated levels of CaMKII may reflect adaptive modulation of synaptic plasticity, potentially influencing the capacity for synaptic adaptation in stress-related cognitive and emotional processes. The coordinated upregulation of PP2A-Aα and CaMKIIα reflects a dynamic regulation of kinase–phosphatase balance within synaptosomes, which may help preserve synaptic function and plasticity under conditions of CSIS.

### 4.3. Cytoskeletal Remodeling in Synaptosomes

Tropomyosin alpha-3 (TPM3) was upregulated in CSIS synaptosomes. TPM3 is an actin-binding cytoskeletal protein that binds F-actin with high affinity [144] and regulates the binding of actin-interacting proteins such as myosin and cofilin [145], thereby modulating actin dynamics critical for neuronal structure and function. Given that cytoskeletal dynamics are fundamental to synaptic plasticity, including dendritic spine morphology, receptor trafficking, and vesicle mobilization, elevated TPM3 protein levels may reflect active cytoskeletal remodeling at synapses in response to CSIS. Alternatively, it could reflect compensatory responses to counteract stress-related dendritic atrophy and spine loss documented in animal models of chronic stress [146].

### 4.4. Coordinated Regulation of Synaptic Protein Synthesis and Degradation

CSIS synaptosomes exhibited upregulation of elongation factor 1-gamma (EEF1G), indicating alterations in the protein synthesis machinery. EEF1G facilitates the elongation phase of translation by delivering aminoacyl-tRNAs to ribosomes [147]. Its elevated protein levels suggest increased local protein synthesis capacity, a critical process for synaptic function and activity-dependent synaptic plasticity, including the production of receptors, scaffolding proteins, and signaling molecules at activated synapses [148].

In parallel, polyubiquitin-C (UBC) was upregulated, suggesting increased protein turnover by the ubiquitin–proteasome system (UPS), which tags proteins for selective degradation by the 26S proteasome. Previous synaptoproteomic studies in rat models of depression have identified the UBC as a central hub in protein–protein interaction networks associated with synaptic function and signaling pathways in both the prefrontal cortex and hippocampus [149], highlighting its potential role in adaptive synaptic remodeling and plasticity [150]. Local UPS-mediated degradation at synapses fine-tunes synaptic protein composition, regulates receptor trafficking, and participates in LTP and LTD processes by selectively degrading synaptic proteins and modulating signaling cascades [151]. The coordinated upregulation of both EEF1G and UBC may reflect a balanced modulation of protein synthesis and targeted degradation of damaged or misfolded proteins, suggesting that CSIS triggers adaptive adjustments in synaptic proteostasis to preserve synaptic function under stress. Such proteostatic remodeling is consistent with the concept that synaptic adaptation to chronic environmental challenges requires dynamic control over the synaptic proteome [152].

### 4.5. Vesicle Trafficking and Synaptic Vesicle Recycling

Hippocampal synaptosomes from CSIS rats exhibited upregulation of clathrin light chain B (CLTB) [17]. CLTB is a neuronal component of the clathrin-mediated endocytosis machinery, essential for synaptic vesicle recycling following neurotransmitter release [153] and the maintenance of synaptic vesicle pools required for sustained neurotransmission [154]. Its upregulation may reflect enhanced synaptic activity or a compensatory adjustment of vesicle recycling to preserve neurotransmitter release under CSIS. Additionally, elevated CLTB levels could facilitate activity-dependent receptor internalization, as CME also mediates the endocytosis of post-synaptic receptors such as AMPA and NMDA receptors, thereby contributing to synaptic plasticity and homeostatic regulation [155]. These findings align with evidence that chronic stress broadly affects vesicle cycling dynamics. Studies using chronic mild stress models have reported differential regulation of synaptic exocytosis and endocytosis components, even though clathrin itself was not specifically quantified in that dataset [133], indicating that vesicle recycling and endocytic processes are sensitive to chronic stress exposure.

## 5. Conclusions

This review demonstrates that CSIS induces distinct, compartment-specific proteomic alterations in hippocampal mitochondrial subpopulations, revealing divergent stress-related responses between NSM and synaptosomes (synaptic mitochondria), which provide insight into the molecular mechanisms underlying stress-induced neurobiological changes. NSM display signatures of metabolic dysfunction, including dysregulated TCA cycle activity, impaired OXPHOS complex expression, and compromised ATP generation. These bioenergetic deficits are accompanied by disrupted mitochondrial transport systems, perturbed protein quality control mechanisms, and MAO-A upregulation, directly linking mitochondrial dysfunction to the monoaminergic dysregulation. Conversely, synaptosomes including synaptic mitochondria demonstrate adaptive remodeling, with enhanced local energy metabolism, activated stress response pathways, and coordinated changes in synaptic protein synthesis and vesicle trafficking that likely preserve synaptic function under stress conditions.

These findings highlight the preferential vulnerability of NSM to chronic stress and suggest that NSM-specific mitochondrial dysfunction may be a key contributor to stress-induced behavioral phenotypes, whereas synaptosomes display proteomic changes suggestive of compensatory adaptation. Understanding these compartmentalized mitochondrial responses not only advances our mechanistic understanding of stress-related psychiatric disorders but also identifies potential therapeutic targets. Future interventions aimed at restoring NSM bioenergetic capacity and proteostasis, while supporting adaptive synaptic mitochondrial responses, may offer more precise and effective treatment strategies for depression.

## Figures and Tables

**Table 1:** Comparative features of synaptic and non-synaptic mitochondria (NSM) with respect to bioenergetics, structural dynamics, stress resilience, and monoamine oxidase (MAO) isoform distribution. Reduced/vulnerable—the feature is decreased or the mitochondrion is more susceptible to stress; maintained/stable—the feature is preserved, indicating resilience and functional stability; increased/activated—the feature is enhanced, e.g., increased fission or sensitivity to stress. Abbreviations: ↑ indicates upregulation; ↓ indicates downregulation; dynamin-1-like protein (DNM1L); hydrogen peroxide (H_2_O_2_); monoamine oxidase A (MAO-A); monoamine oxidase B (MAO-B); mitochondrial calcium uniporter (MCU); mitofusin 1 and 2, fusion proteins (MFN1/2); optic atrophy 1 (OPA1); oxidative phosphorylation (OXPHOS); reactive nitrogen species (RNS); reactive oxygen species (ROS); regulator of mitochondrial calcium uptake (MICU1); transcription factor A, mitochondrial; regulates mtDNA maintenance (TFAM).

Feature	Synaptic Mitochondria	Non-Synaptic Mitochondria (NSM)	References
OXPHOS subunits	Reduced expression (Complexes I, II, IV; COX4I2 exception with higher expression)	Higher/stable expression across OXPHOS complexes	[34]
ATP production	Lower per mitochondrion; requires higher mitochondrial density at synaptic terminals	Higher/stable ATP output per mitochondrion	[34]
Fission/fusion balance	Increased fission (DNM1L ↑), reduced fusion (OPA1 ↓, MFN1/2 ↓); enhanced fragmentation	Balanced fission–fusion dynamics	[34]
Calcium handling	MCU ↓, MICU1 ↓; more sensitive to Ca^2+^ overload	Robust MCU/MICU1 expression; better Ca^2+^ regulation	[27,34,40,41]
Susceptibility to oxidative/ nitrosative stress	High; sensitive to ROS/RNS and lipid peroxidation	Low; more resistant to oxidative challenge	[39,42]
mtDNA maintenance	TFAM ↓; increased mtDNA deletions, reduced transcription	TFAM maintained; stable mtDNA	[34,43]
MAO-A	Present at synaptic terminals, MAO-A catabolizes norepinephrine, serotonin, and dopamine, generating H_2_O_2_. Despite its relatively low abundance in dopaminergic neurons, its oxidative contribution is disproportionate, as it synergizes with the limited local antioxidant capacity to cause oxidative damage	The predominant MAO isoform in neuronal cell bodies and astrocytes, responsible for extracellular serotonin and norepinephrine clearance. H_2_O_2_ generated by astrocytic MAO-A is effectively buffered by glial antioxidant systems, contributing to physiological redox signaling rather than oxidative damage	[44,45,46,47,48]
MAO-B	Predominant at dopaminergic synaptic terminals, catabolizing dopamine, phenylethylamine, and benzylamine with the highest specific activity in synaptosomal fractions. Anchored to the outer mitochondrial membrane, MAO-B oxidizes substrates via a FAD-dependent mechanism, generating H_2_O_2_ as a byproduct	Enriched in astrocytic and serotonergic NSM, MAO-B operates at lower activity than at synaptic terminals. However, chronic low-level H_2_O_2_ production may cumulatively erode glial antioxidant reserves over time	[44,47,48,49,50]
Functional consequence	Vulnerable to energy failure; compromised synaptic plasticity and neuronal integrity	Resilient; preserved energy homeostasis	[34,39,43]

**Table 2:** Protein expression changes in mitochondrial TCA cycle enzymes in stress and depression models: proteomic and biochemical evidence from rat brain studies.

Protein/ Function	Stress Model/Brain Region	Expression Change	Technique	Key Finding	References
DLAT Dihydrolipoyllysine-residue acetyltransferase (PDC E2)	Prenatal stress; rat frontal cortex; total mitochondria	↑	2D-LC-MS/MS	Fluoxetine may positively modulate mitochondrial energetics via the pyruvate dehydrogenase pathway	[65]
	CSIS-resilient vs. control rats; hippocampus; NSM	↑	1D-LC-MS/MS	Enhanced capacity to channel glycolytic intermediates into the TCA cycle, increasing substrate availability for energy production	[66,67]
	Fluoxetine-treated control vs. control rats; hippocampus; NSM;	↑	1D-LC-MS/MS	Fluoxetine directs energy metabolism towards the citric acid cycle and oxidative phosphorylation	[68]
	CSIS; rat; PFC; NSM	↓	LC-MS/MS	An adaptive response of the cells to increased energy demands	[15]
	CMS–depression-susceptible, anxiety-susceptible, stress-resilient (insusceptible) rats; whole hippocampal lysate	↑ all groups; WB confirmed in insusceptible rats	iTRAQ-based proteomics	Western blot-confirmed upregulation of DLAT in the insusceptible group, despite the iTRAQ trend observed across all groups, highlights DLAT as a specific molecular marker of stress resilience	[69]
DLD Dihydrolipoyl dehydrogenase (PDC E3/OGDHC/BCKDHC)	Fluoxetine-treated control vs. control rats; hippocampus; NSM	↑	1D-LC-MS/MS	Fluoxetine directs energy metabolism towards the citric acid cycle and oxidative phosphorylation	[68]
	CMS–depression-susceptible; anxiety-susceptible; stress-resilient rats; whole hippocampal lysate	↑ all groups; WB: anxiety-Sus.	iTRAQ-based proteomics	Enhanced flux of pyruvate into the TCA cycle as a metabolic signature of anxiety	[69]
DLST Dihdrolipoamide S-succinyltransferase (OGDHC-E2)	Simulated microgravity; tail suspension; rat; hippocampus; mitochondria	↑	TMT-based LC-MS/MS	Represents a potential mechanism of cognitive impairment under spaceflight conditions	[70]
	CMS; rat; cerebellum	↑	iTRAQ-based proteomics	Compensatory response to the energy deficit induced by depression	[71]
OGDH 2-Oxoglutarate dehydrogenase E1 (OGDHC E1)	CMS–depression-susceptible; anxiety-susceptible; stress-resilient rats; whole hippocampal lysate	↑ OGDHL, stress-insusceptible rats, confirmed by WB	iTRAQ-based proteomics	OGDHL, a homolog of OGDH, upregulated in stress-resilient rats, upregulation of the TCA cycle as a signature of resilience	[69]
	OGDHC inhibition model (SP/TESP); rat; cerebral cortex	Biphasic	Enzyme activity assay (spectrophotometric)	Biphasic response of the rat brain to progressive OGDH inhibition; compensatory OGDHC upregulation at low inhibitor doses, while higher doses disrupted the glutathione redox state and elevated anxiety-like behavior	[72]
	Simulated microgravity; tail suspension; rat; hippocampus; mitochondria	↑	TMT- based-LC-MS/MS	OGDHC pathway upregulated as part of coordinated TCA activation (including ACO2, CS)	[70]
CS Citrate synthase	Simulated microgravity; tail suspension; rat; hippocampus; mitochondria	↑	TMT- based-LC-MS/MS	CS upregulated, TCA activation under microgravity stress	[70]
	mTBI/sTBI (closed-head impact acceleration); rat whole brain	↓ (sTBI) after 48 and 120 h post injury	ELISA kits	Reflects the progressive collapse of mitochondrial energetics as part of secondary brain injury	[73]
	CMS; rat; cerebellum	↑	iTRAQ-based proteomics	TCA cycle enzymes generally upregulated in CMS cerebellum, compensatory response to energy deficit	[71]
MDH2 Malate dehydrogenase	CSIS vs. control rats; hippocampus; synaptosome	↑	1D-LC-MS/MS	Enhanced mitochondrial TCA cycle capacity	[74]
	Oxidative stress; HT22 hippocampal neurons; in vitro; H_2_O_2_	↑	Spectrophotometric, RT-qPCR	MDH2 activity and mRNA upregulated via miR-743a under H_2_O_2_-induced oxidative stress	[75]
GOT2 Aspartate amino transferase	CSIS-resilient vs. susceptible; rat; hippocampus; NSM	↓ (resilient)	1D-LC-MS/MS	Reduced amino acid-mediated anaplerotic TCA replenishment, favoring direct glycolytic–oxidative energy flux, underlies stress resilience	[66,67]

Literature findings of proteins involved in pyruvate metabolism and TCA cycle enzymes represent data from whole tissue lysates, total mitochondrial fractions or compartment-specific mitochondria in different animal models. Each entry reflects the direction and context of protein expression change under the indicated experimental condition. ↑ indicates upregulation; ↓ indicates downregulation; biphasic indicates a dose-dependent bidirectional response. Where iTRAQ (Isobaric tags for relative and absolute quantitation) proteomics and WB (western blot) data are both available, the WB result is specified separately, as it reflects statistically validated protein-level confirmation. Abbreviations: 2-Oxoglutarate dehydrogenase complex (OGDHC); branched-chain α-keto acid dehydrogenase complex (BCKDHC); chronic mild stress (CMS); chronic social isolation stress (CSIS); liquid chromatography–tandem mass spectrometry (LC-MS/MS); mild traumatic brain injury (mTBI); non-synaptic mitochondria (NSM); 2-Oxoglutarate dehydrogenase-like (OGDHL); prefrontal cortex (PFC); pyruvate dehydrogenase complex (PDC); severe traumatic brain injury (sTBI); succinyl phosphonate (SP); tandem mass tag (TMT); tricarboxylic acid cycle (TCA); triethyl succinyl phosphonate (TESP).

**Table 4:** Mitochondrial transport and carrier proteins with altered expression in depression- and stress-related models.

Protein (Mitochondrial Localization)	Stress Model/Brain Region	Expression Change	Technique	Key Finding	Reference
TOM70 (OMM)	Fluoxetine-treated control rats; hippocampus; NSM	↑	1D-LC-MS/MS	Increased mitochondrial protein import and adaptive mitochondrial remodeling after antidepressant treatment	[68]
VDAC1 (OMM)	CSDS; mouse; hippocampal microglia	↑	Immunofluorescence	Upregulation of VDAC1 associated with mitochondrial stress and depressive-like behavior	[114]
VDAC1/VDAC2 (OMM)	Zinc-deficient rats; PFC	↑/↑	LC-MS/MS	Altered mitochondrial membrane permeability and metabolic dysfunction	[101]
VDAC2 (OMM)	CSIS; PFC; NSM	↑	LC-MS/MS	Upregulation VDAC2 indicates metabolic adaptation to chronic stress	[15]
VDAC1 (OMM)/ PiC (IMM)	CSIS; rat; hippocampus; synaptosomal mitochondria	↓/↓	LC-MS/MS	Downregulation in CSIS-resilient vs. CSIS-susceptible (VDAC1, PiC) and CSIS-resilient vs. control (VDAC1) rats suggests altered mitochondrial transport linked to stress resilience	[74]
VDAC2 (OMM)/ PiC (IMM)	CSIS; rat; hippocampus; NSM	↓/↑/↓	LC-MS/MS	Differential regulation (CSIS-resilient vs. CSIS-susceptible VDAC2 ↓, PiC ↑ and CSIS-resilient vs. control PiC ↓) indicates mitochondrial metabolic adaptation between resilient and susceptible/control rats	[66]
PiC (IMM)	CMS; rat; hippocampus	↑	iTRAQ-based proteomics	Upregulation in CMS-insusceptible rats suggests adaptive mitochondrial phosphate transport	[69]
PiC (IMM)/VDAC2 (OMM)	CSIS-resilient vs. CSIS-susceptible rats; hippocampus; NSM	↑/↓	1D-LC-MS/MS	Enhanced mitochondrial energy metabolism and reduced mitochondrial permeability compared with stress-susceptible animals	[66]
ANT1/ANT2 (IMM)	CMS; rat; hippocampus; depression- and anxiety-susceptible and insusceptible rats	↑	iTRAQ-based proteomics	Altered mitochondrial ATP/ADP exchange and energy metabolism in depression-susceptible rats	[69]
2-OGC (IMM)	Human ischemic brain	↓	iTRAQ-based proteomics	Downregulation of 2-OG transport protein levels suggests impaired metabolic coupling	[115]
2-OGC/PiC (IMM)	CMS; rat; hippocampal synaptosome (synaptic mitochondria)	↔/↑	iTRAQ-based proteomics	Significant changes in synaptic mitochondrial proteins implying disruption of oxidative phosphorylation and transport systems	[97]
GC1 (IMM)	Mouse cortex; MitoQ responders vs. non-responders	↓	WB	Downregulation associated with anxiolytic response to mitochondrial antioxidant treatment	[116]
SFXN-1 (IMM)	Alzheimer’s disease; brain cortex	↓	iTRAQ-based proteomics	Disrupted mitochondrial amino-acid metabolism	[117]
SFXN-3 (IMM)	Sfxn3-KO mice brain synaptosomes	Dysregulated	TMT- LC-MS/MS	Regulates levels of proteins known to be associated with neurodegeneration and cell death pathways	[118]

Literature findings of protein expression changes in mitochondrial transport and carrier proteins across rodent stress models (CSIS, chronic social isolation; CSDS, chronic social defeat stress; CMS, chronic mild stress; zinc deficiency, ischemia) and human post-mortem brain tissue (Alzheimer’s disease, ischemic brain injury), as identified by mass spectrometry-based proteomics, western blot, and immunofluorescence. Proteins are grouped by mitochondrial membrane localization (OMM, outer mitochondrial membrane; IMM, inner mitochondrial membrane). Regulation is indicated as upregulation (↑), downregulation (↓), no significant change (↔), or dysregulation without specified directionality. VDAC, voltage-dependent anion channel; ANT, adenine nucleotide translocator; PiC, phosphate carrier; PFC, prefrontal cortex; 2-OGC, 2-oxoglutarate/malate carrier; GC1, glutamate carrier 1; SFXN, sideroflexin; NSM, non-synaptic mitochondria; WB, western blot.

**Table 5:** Changes in the protein expression of heat shock proteins (HSP60/HSP90 family) in preclinical and clinical models of depression.

Protein	Stress Model/Brain Region	Expression Change	Technique	Key Finding	Reference
HSP60 (HSPD1)	Microglia-specific KO mice; whole brain	↓ (KO)	Nano-LC–MS/MS	Loss of HSP60 induces depression-like behavior, synaptic loss, and microglial overactivation → impaired glutamatergic signaling	[126]
HSP60 (HSPD1)	Social defeat stress; mouse; hippocampus	↓	WB	Downregulation of hippocampal HSP105 is associated with depression-like phenotype	[124]
HSP60 (HSPD1)/HSP10 (HSPE1)	CSIS-resilient vs. control rats; hippocampus; NSM	↓	1D-LC-MS/MS	Reduced mitochondrial stress response and more efficient protein folding observed in CSIS-resilient rats	[66]
HSP10 (HSPE1)	Inferred from mitochondrial proteostasis impairment; not directly measured	↓	Indirect evidence	HSP60 deficiency causes downregulation of mitochondrial proteome, implying HSPE1 dysfunction in stress conditions	[127]
HSP90-β (HSP90AB1)	CMS; rat; hippocampus, mRNA	↑	RT-qPCR	Upregulated as part of an adaptive stress response; linked to neuroprotection and enhanced protein folding capacity	[125]
HSP90-α (HSP90AA1)	Human MDD (non-psychotic); DLPFC	↑	1D-LC-MS	Stress-induced protein folding response	[95]

Changes in the expression of heat shock proteins (HSP60/HSP90 family) in preclinical and clinical models of depression. Regulation is indicated as upregulation (↑), downregulation (↓); ↓ KO, loss of function due to genetic knockout; ↓ indirect, change inferred indirectly, without direct measurement. CMS, chronic mild stress; CSIS, chronic social isolation stress; MDD, major depressive disorder; KO, knockout; DLPFC, dorsolateral prefrontal cortex; RT-qPCR, real-time quantitative PCR (mRNA expression analysis); 1D-LC-MS/MS, one-dimensional liquid chromatography coupled with tandem mass spectrometry (proteomic analysis); WB, western blot.

## Data Availability

No new data were created or analyzed in this study. Data sharing is not applicable to this article.

## Supplementary Materials

The following supporting information can be downloaded at https://www.mdpi.com/article/10.3390/ijms27083386/s1.
